# Temporal-Difference Reinforcement Learning with Distributed Representations

**DOI:** 10.1371/journal.pone.0007362

**Published:** 2009-10-20

**Authors:** Zeb Kurth-Nelson, A. David Redish

**Affiliations:** Department of Neuroscience, University of Minnesota, Minneapolis, Minnesota, United States of America; Indiana University, United States of America

## Abstract

Temporal-difference (TD) algorithms have been proposed as models of reinforcement learning (RL). We examine two issues of distributed representation in these TD algorithms: distributed representations of belief and distributed discounting factors. Distributed representation of belief allows the believed state of the world to distribute across sets of *equivalent states*. Distributed exponential discounting factors produce hyperbolic discounting in the behavior of the agent itself. We examine these issues in the context of a TD RL model in which state-belief is distributed over a set of exponentially-discounting “micro-Agents”, each of which has a separate discounting factor (*γ*). Each µAgent maintains an independent hypothesis about the state of the world, and a separate value-estimate of taking actions within that hypothesized state. The overall agent thus instantiates a flexible representation of an evolving world-state. As with other TD models, the value-error (*δ*) signal within the model matches dopamine signals recorded from animals in standard conditioning reward-paradigms. The distributed representation of belief provides an explanation for the decrease in dopamine at the conditioned stimulus seen in overtrained animals, for the differences between trace and delay conditioning, and for transient bursts of dopamine seen at movement initiation. Because each µAgent also includes its own exponential discounting factor, the overall agent shows hyperbolic discounting, consistent with behavioral experiments.

## Introduction

Temporal-difference (TD) learning algorithms have been proposed to model behavioral reinforcement learning (RL) [1]–[3]. The goal of reinforcement learning is to learn what actions to select in what situations by learning a value function of situations or “states” [4]. (As noted by Daw *et al*. [5], it is not necessarily true that the agent's estimate of the world-state always corresponds to the actual state of the world. We have already explored some of the potential consequences of this mismatch in another paper [6] and will not address it here.) In TD models, the value function is learned through the calculation of a value-prediction error signal (termed 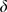, [4], [7], [8]), calculated each time the agent changes world-states. 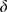 reflects the difference between the value-estimate and the actual value (including immediate reward) observed on the transition. From 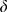, the value-estimate of the old state can be updated to approach the observed value. This 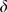 signal appears at unexpected rewards, transfers with learning from rewards to anticipatory cue stimuli, and shifts with changes in anticipated reward [4], [8]. This algorithm is a generalization of the early psychological reward-error models [9], [10]. Components of these models have been proposed to correspond to neurophysiological signals [1], [2], [8], [11]–[14]. In particular, the firing of midbrain dopaminergic neurons closely matches 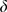.

TD RL models have been able to provide strong explanations for many neurophysiological observations, such as qualitative changes in dopamine firing [1], [5], including changes at first thought not to reflect prediction error (e.g. generalization and exploration [15]). More recent experiments have shown quantitative matches to the predictions of these models [16]–[22]. In addition, more recent models have been based on distributed representations of belief within those state-spaces [5], [23]–[26].

In this paper, we examine the effects of distributed state representation, distributed value-representation, and distributed discounting rate in TD learning.

- Distributed discounting rates along with distributed value representation lead to hyperbolic discounting, matching the hyperbolic discounting experimentally observed in humans and animals.
- Distributed representations of state-belief allow the agent to divide its believed state across multiple *equivalent states*. This distributed state-representation can account for the slowing of learning rates across intertrial intervals and trace conditioning paradigms, and can account for dopamine signals seen at movement initiation in certain instrumental conditioning paradigms.

These two hypotheses are separable and produce separable predictions, but together they form a coherent and parsimonious description of a multi-micro-agent (µAgent) TD model of reinforcement learning that provides a good fit to the experimental data. We will make clear in the simulations below which components are necessary for which results, and in the discussion which predictions follow from which hypotheses.

This multiple micro-agents model is consistent with anatomical studies suggesting that the basal ganglia consist of separable “loops” that maintain their separation through the basal ganglia pathway [27]–[29]. The model is also consistent with recent fMRI studies suggesting that the striatum consists of functional “slices” reflecting a range of discounting factors [30], [31].

## Methods

It is important to note that the theoretical consequences of distributed representation are independent of many of the methodological details. However, in order to implement simulations, specific choices have to be made. Throughout the methods section, we will identify which simulation details are theoretically important and which are not.

**Figure 1:**
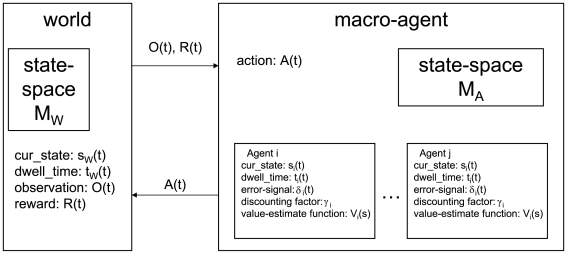
Model overview. The world communicates with the agent by sending observations and rewards and receiving actions. The world maintains its own “true” state and dwell time in that state. The agent is composed of independent µAgents that each maintain a belief of the world's state and dwell time. Each µAgent has its own value estimate for each state and its own discounting factor, and generates an independent *δ* signal. The µAgents' belief is integrated for action selection by a voting process.

The simulation comprised two entities: the world and the agent. The world consisted of a semi-Markov state space (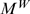) with two additions. First, it provided *observations* and *rewards* to the agent; second, its current state could be changed by an *action* of the agent. The agent consisted of a set of µAgents, each of which contained a model of the world 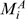, a hypothesis of the state of the world 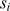, a value function of those states 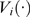, and an exponential discounting factor 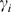. On each time step, a value-prediction-error 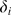 was calculated independently by each µAgent. The overall agent performed actions based on the state beliefs and value functions of the µAgents, and the 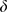 signals of all µAgents could be averaged to represent an overall 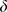 signal. The world and agent were simulated in discrete time-steps. The world provided an observation or null-observation to the agent on each time-step, and the agent provided an action or null-action to the world on each time-step. See Figure 1 and Table 1 for an overview of the model structure.

**Table 1:** Variables and parameters used in the simulations.

Variables	
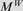	world model
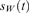	current world state
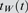	current dwell time in 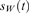
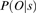	probability of observing observation 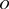 given state 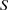
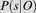	calculated from 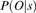
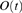	observation passed from world to macro-agent at time 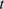
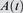	action passed from macro-agent to world at time 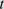
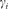	discounting factor, 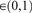 for µAgent 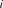
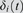	value-prediction-error for µAgent 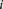
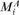	µAgent world model ( 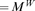 ) for µAgent 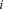
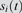	hypothesized state for µAgent 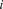
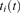	hypothesized dwell time in 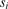 for µAgent 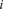
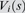	value function for µAgent 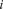

### State-space/process-model

Both the world and the agent contain an internal state-space: 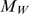 and 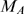, respectively. In principle it is not necessary that 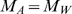. In fact, it is quite possible for each µAgent to have an individual world-model 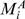. In the simulations used, all µAgents used an identical state-space model 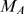, defined as identical to the world-model 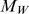.

States corresponded to temporally extended circumstances salient to the agent, such as being located at an arm of a maze or waiting within an interstimulus interval. Transitions defined jumps from one state to another. On entry into a state, a random time was drawn from that state's dwell-time distribution, which determined how long the world would remain within that state before a transition occurred. Observations provided feedback from the world to the agent on each time-step and were drawn from the 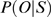 distribution, dependent on the actual state of the world 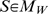. Rewards were a special type of observation, which included a magnitude and were used in the calculation of 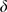.

### The world

The world consisted of a semi-Markov state process, a current state 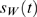, a dwell-time within that state 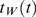, a current observation 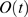, and a current reward 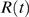. Only observation (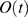) and reward (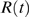) were provided to the agent.

A transition in the state of the world could occur due to a process inherent in the world or due to the action of the agent. For example, in our model of the adjusting-delay assay, the world will remain in the action-available state (providing an *observation* of two levers to the animal) until the agent takes an action. In contrast, once the agent has taken an action and the world has transitioned to one of the delay states (ISI-1, or ISI-2), the world will remain in that state for an appropriate number of time-steps and then transition to the reward state, irrespective of the agent's actions.

### The macro-agent

The macro-agent corresponded to the animal or traditional “agent” in reinforcement learning models. The macro-agent interacted with the world and selected *actions*. Internal to the macro-agent were a set of 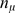 µAgents, which instantiated the macro-agent's belief distribution of the state of the world. Smaller 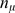 yielded noisier output. However, results were qualitatively unchanged down to 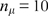 = 10. Results were stabler with explicitly uniform distributions of 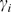. The only simulation in which this made a noticeable difference was in the measure of hyperbolic discounting (because the hyperbolic function emerges from the sum of many exponentials).

### Individual µAgents

Each µAgent 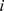 was fully specified by a five-tuple 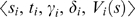, encoding the µAgent's currently believed state, 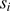; the believed dwell-time, 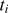 (i.e., how long since the last state transition), the µAgent's internal discounting parameter 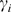, the current value-prediction-error signal 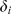, and the µAgent's value estimation function 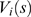. Each µAgent contained its own individual discounting parameter 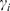, drawn from a uniform random distribution in the range 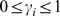.

The state, 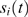, and dwell-time, 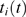, of each µAgent are hypotheses of the actual state of the world, 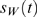, and the actual dwell-time, 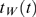 of the world within that state. Even if the µAgent knew the true initial state of the world, that hypothesis could diverge from reality over time. In order to maintain an accurate belief distribution, µAgents at each time-step computed the probability 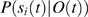, where 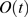 was the observation provided by the world at time 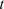, and 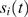 was µAgent 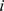
's state at time 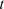. µAgents with low 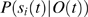 updated their state belief by setting 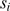 to a random state 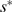 selected with probability 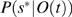. This is one of three mechanisms by which µAgents could change state (see below). An individual 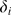 value error signal was computed at each µAgent state transition (see below).

### Action selection

Actions can only occur at the level of the macro-agent because they are made by the organism as a whole. Because the state belief and value belief are distributed across the µAgents, a mechanism was required to select the best action given that belief distribution. In the model as implemented here, the macro-agent simply “took a vote” from the µAgents as to which action to perform. Each µAgent provided an equally-weighted measure of the expected value for each action. The exact action selection algorithm is not crucial but must take account of the belief distribution and must balance exploration and exploitation.

Actions were selected based on an 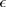-greedy algorithm [4], with 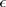 decreasing with each trial. This produces exploration early and exploitation later. At each time-step, a random number was drawn between 0 and 1. If that number was less than 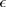, then actions were taken based on the µAgents' vote on what actions were possible. If the number was greater than 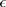, then actions were taken based on the µAgents' vote on the expected values of the subsequent states. 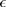 started at 1 and was multiplied by a factor of 0.95 each time reward was delivered, producing an exponential decrease in exploration with experience.

#### Exploration

If the macro-agent decided to explore, the action to be taken was drawn from a distribution based on which actions the µAgent population suggested was possible.
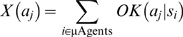
(1)where 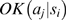 was true (1) if action 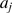 was available from µAgent 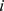
's believed state 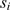 and false (0) otherwise. Actions were then selected linearly from the distribution of possible actions:
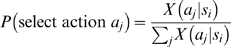
(2)


#### Exploitation

If the macro-agent decided to exploit the stored value functions, then actions were selected based on the normalized expected total value of the achieved state:
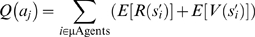
(3)where 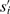 the state that would be achieved by taking action 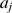 given the current state 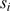 of µAgent 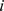, 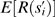 the expected reward in state 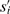, 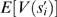 the expected value of state 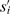. 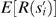 was calculated from the internal world model 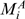, and 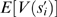 was calculated from the internal value representation stored in µAgent 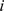. If action 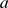 was not available from the current state of µAgent 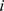, µAgent 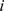 was not included in the sum. Because our simulations only include reinforcement, only positive transitions were included, thus 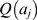 was rectified at 0. (Our simulations only include reinforcement primarily for simplicity. The mechanisms we describe here can be directly applied to aversive learning; however, because the extinction literature implies that reinforcement and aversion use separate, parallel systems [6], we have chosen to directly model reinforcement here.) Actions were then selected linearly between the possible 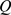 functions:
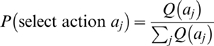
(4)


Once an action was selected (either from 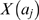 or from 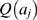), a decision was made whether to take the action or not based on the number of µAgents who believed the action was possible:
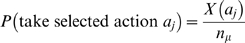
(5)


If the selected action was taken, the agent passed action 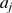 to the world. If the selected action was not taken, the agent passed the “null action” (which did not change the state and was always available) back to the world. If the macro-agent tried to take action 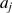, but action 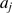 was incompatible with the actual world state 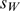, no action was taken, and the “null action” was provided to the macro-agent.

When proportions of actions were measured (e.g. in the discounting experiments), proportions were only measured after 200 trials (by which time 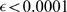).

### µAgent transitions

There were three possible mechanisms by which µAgents could make transitions between hypothesized belief states 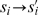.

1. **Internal transitions.** On each time-step, each µAgent 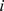 decided whether to transition or not as a function of the dwell-time distribution, given its hypothesized state 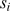 and its hypothesized dwell-time 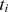. If the µAgent took a transition, it followed the transition matrix stored within 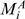.
2. **Taking an action.** If the macro-agent took action 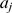, providing 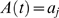 to the world, all µAgents were then updated assuming the action occurred given the state-hypothesis of the µAgent 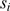. If the action was incompatible with the µAgent's state belief, the µAgent's belief-state 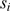 was revised as described below.
3. **Incompatible observations.** On each time step, each µAgent 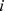 compared the observation provided by the world 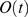 with the observation expected given its internal hypothesized state 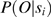. If 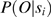 was 0 (meaning the observation was incompatible with 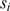), the µAgent transitioned to a new state based on the probability of the state given the current observation 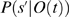.

### Calculating the error signal: *δ*

µAgents could experience a state transition as a consequence of the macro-agent taking an action, as a consequence of its dwell-time belief, or as a consequence of revising its state hypothesis due to low fitness. No matter how the µAgent changed its state hypothesis 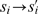, when µAgent 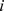 made a transition, it generated a 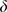 contribution 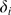 according to
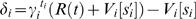
(6)where 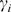 was the discounting parameter of the µAgent, 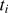 was the µAgent's hypothesized time since the last transition, 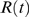 was the observed reward at time 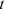, 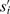 was the new state hypothesis to which the µAgent transitioned, and 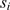 was the old state hypothesis from which the µAgent transitioned. Of course, the process of transitioning set the µAgent's believed state to be 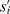 and 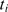 to be 0. Note that 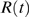 is not a function of 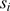, but rather delivered to the agent from the world, based on the world state 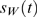. Note that equation 6 is an exponential discounting function. Thus, each µAgent performed exponential discounting. The macro-agent showed hyperbolic discounting as an emergent process from the set of all the µAgents. Also, note that both the value of the new state and the current reward were discounted, as the sum of these quantities represents the total expected value of making a transition to a new state. Thus the sum 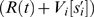 must be discounted proportional to the time the agent remained in state 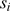 before reaching the new state 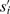.

On each µAgent state transition, the µAgent updated its internal estimation of the value of its hypothesized state 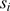, using its individual 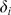:
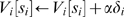
(7)where 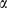 was the learning rate. The mean of the 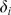 signals 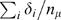 from all µAgents conforms to the quantity reported in this paper as “the 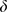 signal of the model” but never appeared explicitly within the simulation code. It is this total 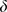 signal, however, which was compared to the population dopamine signal [13], [32]–[35].

## Results

### Hyperbolic discounting

**Figure 2:**
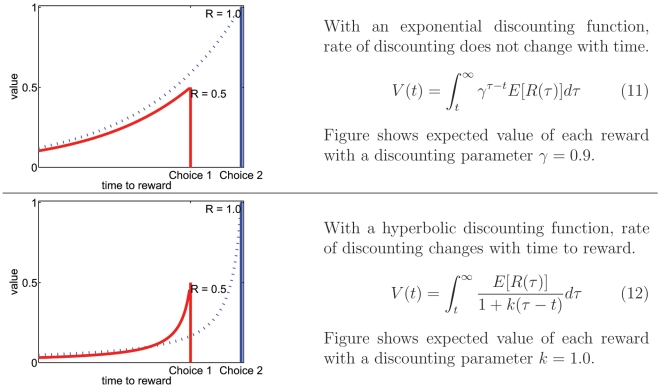
Discounting functions. (A) Exponential discounting reduces value by a fixed percentage over any time interval. Therefore the relative preference of two future rewards does not change as the time to these rewards approaches. (B) In hyperbolic discounting, a later/larger reward may be preferred over a sooner/smaller reward until the rewards draw closer, at which point choice preference can reverse so the sooner/smaller reward is impulsively preferred. After Ainslie [38], [41].

Value, as defined in reinforcement learning models, is the integrated, expected reward, minus expected costs. The longer one must wait for a reward, the more likely it is for an unexpected event to occur, which could invalidate one's prediction [36], [37]. Agents, therefore, should discount future rewards: the more one must wait for the reward, the less valuable it should be. In addition, early rewards are more valuable than late rewards because early rewards can be invested (whether economically or ethologically) [36]–[38]. Any function that decreases with time could serve as a discounting function. In many situations, humans and other animals discount future rewards using a hyperbolic function [38]–[42] matching equation 12 rather than equation 11 (Figure 2).

TD algorithms incrementally learn an estimate of the value function, and thus require either a general analytical solution to the discounting function or an incremental calculation such that the value can be discounted with each timestep [8], [43], [44]. Because the discounting rate changes with time in hyperbolic discounting [38], [41], the calculation cannot be performed incrementally [8]. We suggest a possible mechanism for generating hyperbolic discounting via a multitude of exponential discounting factors. In the limit as the number of exponential discounters (having uniformly distributed discounting factors 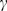) approaches infinity, the average resultant discounting approaches hyperbolic. (See Supporting Information *Appendix S1* for mathematical proof.) In practice, having dozens or more of exponential discounters produces a close approximation to hyperbolic discounting.

Because each µAgent has an independent (exponential) discounting factor but actions are taken by the macro-agent based on a voting process of actions suggested by the µAgents, the macro-agent will show a discounting curve that is the average of all the µAgent discounting curves. If the µAgent discounting curves are exponential functions with 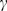 uniformly distributed over the range from 0 to 1, then the macro-agent will show approximately hyperbolic discounting in its behavior. The hypothesis that hyperbolic discounting arises from a (finite) set of exponential factors is consistent with recent fMRI observations [30], [31] and suggests that the difference between this approximate hyperbolic and true hyperbolic discounting could be tested with sufficiently large data sets [45], [46].

#### Simulations

**Figure 3:**
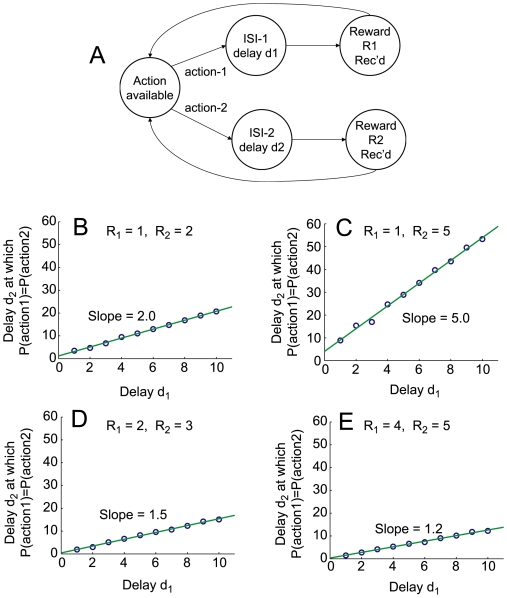
Hyperbolic discounting. (A) State-space used. (B–E) Mazur-plots. These plots show the delay 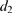 at the *indifference point* where actions 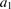 and 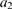 are selected with equal frequency, as a function of the delay 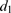. The ratio of actions 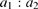 is an observable measure of the relative values of the two choices. Blue circles represent output of the model, and green lines are least-squares fits. For hyperbolic discounting, the slope of the line will equal the ratio 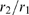, with a non-zero 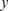-intercept. Compare [39], [40].

In order to measure the effective discounting function of our model, we modified the adjusting-delay assay of Mazur [39]. A five-state state-space was used to provide the macro-agent a choice between two actions, each of which led to a reward. In short, the agent was provided two choices (representing two levers): action 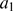 brought reward 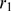 after delay 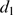 and action 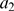 brought reward 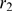 after delay 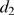. For a given experiment, both rewards 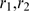 and one delay 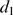 were held fixed, while the other delay 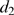 was varied. For each set of 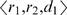, the delay 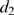 was found where the number of 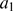 choices taken matched the number of 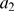 choices taken in 300 trials. At this point, the actions indicate that the two discounting factors in the two delays exactly compensate for the difference in magnitudes of the two rewards. The delay 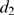 at this equivalent action-selection point can be plotted against different fixed values of 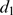. The slope of that curve indicates the discounting function used by the agent [39]. In the case of exponential discounting (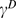 where 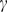 is the discounting factor, 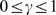, and 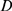 is the delay), the slope will be 1, regardless of 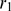 or 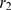. In the case of reciprocal (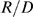) discounting, the slope will equal to the ratio of rewards 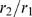, and the 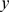-intercept will be 0. In the case of hyperbolic discounting (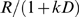, [39], [40], [47]), the slope will equal the ratio 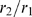, and in the case where 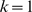, the 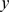-intercept will be 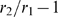. Simulations produced a slope equal to the ratio of rewards 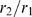 (Figure 3) and a 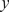-intercept approximating 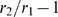, indicating that, even though each individual µAgent implemented an exponential discounting function, the macro-agent showed hyperbolic discounting, compatible with the behavioral literature [39]–[41], [47], [48].

#### Discounting across multiple steps

**Figure 4:**
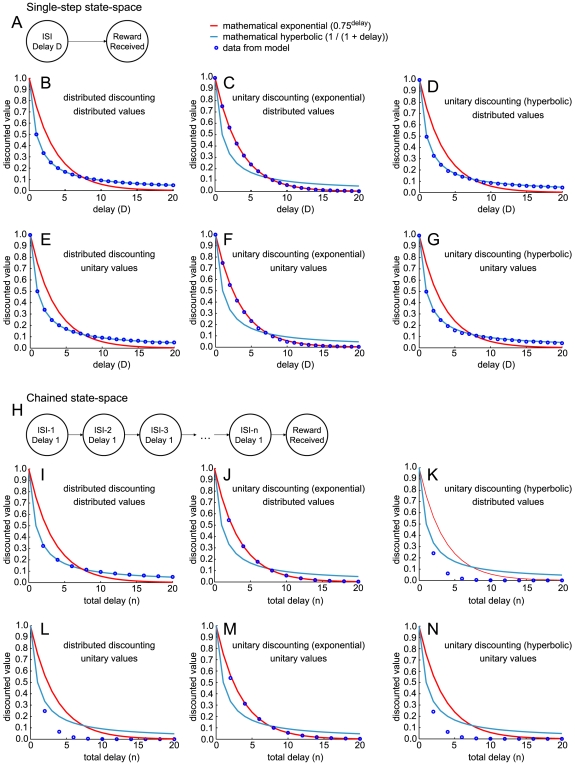
Discounting across state-chains. (A) Single-step state-space used for B–G. (B,E) When the model consists of a set of exponential discounters with *γ* drawn uniformly from (0,1), the measured discounting closely fits the hyperbolic function. (C,F) When the model consists of a single exponential discounter with 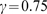, the measured discounting closely fits the function 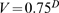 (exponential). (D,G) When the model consists of a single hyperbolic discounter, the measured discounting closely fits the function 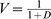 (hyperbolic). (H) Chained state-space used for I–N. (I) If values are distributed so each exponential discounter has its own value representation, the result is hyperbolic discounting over a chained state space. (J,M) A single exponential discounter behaves as in the single-step state space, because multiplying exponentials gives an exponential. (K,N) A single hyperbolic discounter now behaves as an exponential discounter with 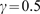, because each step is discounted by 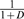 , where 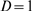. (L) Likewise, a set of exponential discounters with shared value representation behave as an exponential discounter with 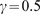, for the same reason.

Temporal difference learning can use any function as a discounting function across a single state-transition. However, if hyperbolic discounting is implemented directly, a problem arises when discounting is measured over a sequence of multiple state transitions. This can be seen by comparing two state-spaces, one in which the agent remains in state 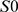 for ten timesteps and then transitions to state 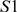 (Figure 4A), and another in which the time taken between state 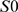 and 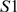 are divided into ten substates, with the agent remaining in each for one timestep (Figure 4H). These two statespaces encode equivalent information over equivalent time and (theoretically) should be discounted equivalently. If temporal discounting were implemented directly with equation 12, then the agent would show hyperbolic discounting across the first statespace, but not the second.

We tested this explicitly by comparing four simulations (see Figure 4):

1. Discounting is not distributed, and 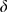 is calculated by
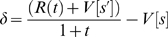
(8)In this condition, the measured discounting of the model was hyperbolic over a single-step state-space (Figure 4G). However, over an equivalent chained state-space (Figure 4N), the macro-agent discounted each state-jump hyperbolically. Since each state had a delay of D = 1, the amount of discounting for each state-jump was 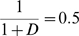, leading to exponential discounting (with 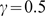) over the chain of states. This occurred whether or not value representation was distributed (Figure 4D,K).
2. Discounting is not distributed, and 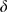 is calculated by
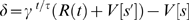
(9)where 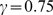. In this condition, the measured discounting of the model was exponential over both the single-step state-space (Figure 4F) and the chained state-space (Figure 4M). This occurred whether or not value representation was distributed (Figure 4C,J).
3. Discounting is distributed (i.e., each µAgent has a different exponential discounting rate 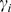 drawn uniformly at random from 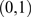). 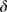 is thus calculated using Eqn. 6 as specified in the Methods section. However, value representation is not distributed; all µAgents access the same value representation 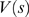. Thus, Eqn. (7) was replaced with
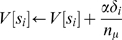
(10)In this equation, although the µAgents could update different states based on their hypothesized state-beliefs, all values were united into a single universal value function 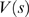. In this condition, the macro-agent reverted to the one-step hyperbolic equation in version 1 (Eqn 8), showing hyperbolic discounting in the single-step state-space (Figure 4E) but not the chained state-space (Figure 4L). In the chained state-space, the sum of distributed exponential discounting rates produces hyperbolic discounting across each state-jump, so across the chain of states discounting was exponential (with 
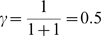
).
4. Both discounting (Eqn. 6) and value (Eqn. 7) are distributed. This model showed hyperbolic discounting under both the single-step state-space (Figure 4B) and the chained state-space (Figure 4I). Because each µAgent has its own value representation for each state, the value decrease across each state-jump was exponential, with each µAgent having a different 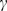. Thus the average value of a state was the average of these exponentially-discounted values, which was hyperbolic.

It is still an open question whether real subjects show differences between single-step and chained state-space representations. Such an experiment would require a mechanism to change the internal representation of the subject (as one state lasting for ten seconds or as ten states lasting for one second each). This could be tested by concatenating multiple delays. Simulation 1, using explicit hyperbolic discounting, predicts that discounting across a chained state-space will be much faster than discounting across a single-step. Whether this occurs remains a point of debate [49]. The model of distributed discounting and distributed values best fits the data that discounting is hyperbolic even across multiple delays.

#### Non-uniform distributions of discounting rates

So far in exploring distributed discounting, we have selected 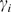 uniformly from 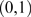. Using this 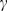 distribution, the overall agent exhibits hyperbolic discounting as 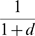. However, different 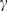 distributions should produce different overall discounting functions.

**Figure 5:**
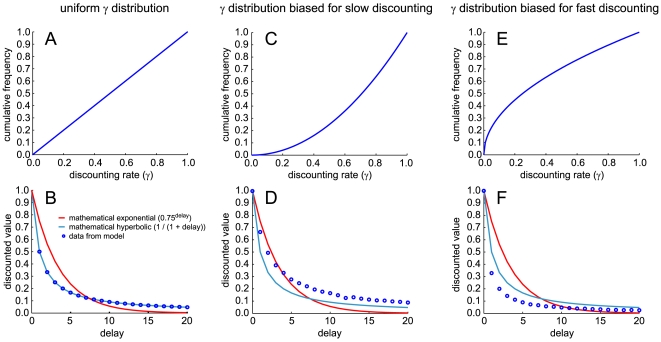
Rate of discounting depends on γ distribution. (A) The uniform distribution of exponential discounting rates used in all other figures. (B) As shown in Figure 4, the overall discounting is hyperbolic. (C) A distribution of exponential discounting rates containing a higher proportion of slow discounters. (D) Overall discounting is slower. (Note that it is now fit by the function 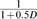.) (E) A distribution of exponential discounting rates containing a higher proportion of fast discounters. (F) Overall discounting is faster. (It is now fit by the function 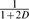.)

We tested this by altering the 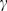 distribution of the µAgents and measuring the resulting changes in discounting of the overall agent. In the uniform distribution (which was also used for all other simulations in this paper), 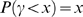, 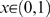 (Figure 5A). As was also shown in Figure 4B, this results in hyperbolic discounting for the overall agent (Figure 5B). Fitting the function 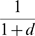 to this curve gives an 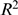 of 0.9999 (using 200 µAgents; the fit improves as 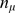 increases). To bias for slow discounting rates, we used the distribution 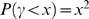 (Figure 5C). The measured discounting of the overall agent using this 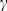 distribution was slower (Figure 5D) and was well-fit by the function 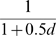. To bias for fast discounting rates, we used the distribution 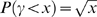 (Figure 5E). The measured discounting of the overall agent using this 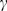 distribution was faster (Figure 5F) and was well-fit by the function 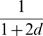. These results match theoretical predictions for the effect of biased 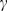 distributions on discounting [37]. Mathematically, it can also be shown that non-hyperbolic discounting can result from 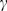 distributions that do not follow 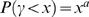; for example if the 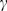 distribution is bimodal with a relative abundance of very slow and very fast discounting µAgents.

Smokers, problem gamblers, and drug abusers all show faster discounting rates than controls [48], [50]–[53]. Whether discounting best-fit by different time-constants is exactly hyperbolic or not is still unknown (see, for example, [48], [51], [54], in which the hyperbolic fit is clearly imperfect). These differences could be tested with sufficiently large data sets, as the time-courses of forgetting have been: although forgetting was once hypothesized to follow hyperbolic decay functions, forgetting is best modeled as a sum of exponentials, not as hyperbolic or logistic functions [45], [46]. Similar experiments could differentiate the hyperbolic and multiple-exponential hypotheses.

All subsequent experiments used a uniform distribution of 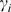.

### Distributed belief

Because each µAgent instantiates an independent hypothesis about the state of the world, the macro-agent can maintain a distributed belief of world-state. We describe two consequences of distributed belief that explain experimental data.

First, some situations contain readily identifiable cues which allow those times when the agent is in those situations to be separated from times when the agent is not. For example, during delay conditioning, there is a specific stimulus (e.g. a light or tone) that is played continuously through the delay. Separating “tone-on” situations from “tone-off” situations readily identifies the inter-stimulus-interval. Other situations are not as readily identifiable. For example, during inter-trial intervals and during the inter-stimulus interval in trace conditioning, there is a gap in which the agent does not know what cues to attend to. Our model simulates this cue ambiguity by representing the gap with a set of identical *equivalent states*. These equivalent states slow value learning because each state only holds a fraction of the µAgent state-belief distribution and therefore only receives a fraction of the total 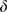 produced by a state-transition. We suggest that equivalent-states explain the well-established slower learning rates of trace compared to delay conditioning [55], and explain the slow loss of dopamine signal at conditioned stimuli with overtraining [32].

Second, distributed belief allows TD to occur in ambiguous state-spaces [5], [6], which can explain the generalization responses of dopamine [15], [34] and the transient burst of dopamine observed at movement initiation [56], [57].

### Trace and delay-conditioning

In delay conditioning, the CS remains on until the reward is delivered, while in trace conditioning there is a gap between the CS and US—the CS disappears before the US appears [55], [58]. This simple change produces dramatic effects: trace conditioning takes much longer to learn than delay conditioning, and requires the hippocampus, unlike delay conditioning [55], [59], [60]. One possible explanation for the difference is that, because there is no obvious cue for the animal to pay attention to, the intervening state representation during the gap in trace conditioning is spread out over many multiple “equivalent states”. (There is new evidence that trace conditioning requires hippocampus only under aversive training conditions [61], which may suggest that other structures can bridge the gap in appetitive trace conditioning. This does not change our primary hypothesis—that trace conditioning entails an “equivalent states” representation of the gap between CS and US.)

Because the µAgents model can represent distributed belief, we can model trace conditioning by placing a collection of equivalent states between the cue and the reward. As noted above, because value learning is distributed across those equivalent states, value is learned more slowly than in well-identified states.

#### Simulations

**Figure 6:**
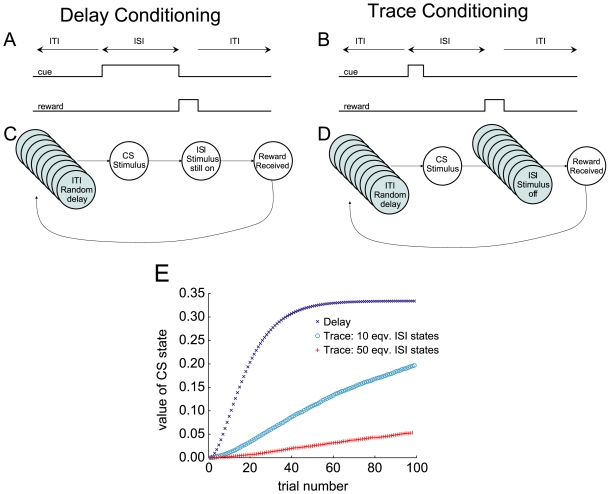
Trace and Delay conditioning paradigms. (A,B) Explanation of delay (A) and trace (B) conditioning. In delay conditioning, the cueing stimulus remains on until the reward appears. In trace conditioning, the cueing stimulus turns back off before the reward appears. (C,D) State spaces for delay-conditioning (C) and trace-conditioning (D). In delay conditioning, the presence of the (presumably salient) stimulus produces a single, observationally-defined state. In trace conditioning the absence of a salient stimulus produces a collection of equivalent states. (E) Simulations of trace vs. delay conditioning. Value learning at the CS state is slower under trace conditioning due to the intervening collection of equivalent states. Larger sets of equivalent states lead to slower value-growth of the CS state.

In order to test the effect of a collection of equivalent states in the inter-stimulus time, we simulated a Pavlovian conditioning paradigm, under two conditions: with a single state intervening between CS and US, or with a collection of 10 or 50 equivalent states between the CS and US. As can be seen in Figure 6, the value of the initial ISI state (when the CS turns on) 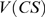 increases more quickly under delay than under trace conditioning. This value function is the amount of expected reward given receipt of the CS. Thus in trace conditioning, the recognition that the CS implies reward is delayed relative to delay conditioning. Increasing the number of equivalent states in the ISI from 10 to 50 further slows learning of 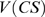 (Figure 6).

#### Discussion and implications

Sets of equivalent states can be seen as a model of the attention the agent has given to a single set of identified cues. Because the stimulus remains on during delay conditioning, the stimulus may serve to focus attention, which differentiates the Stimulus-on state from other states. Because there is no obvious attentional focus in the interstimulus interval in trace conditioning, this may produce more divided attention, which can be modeled as a large collection of equivalent intervening states in the ISI period. Levy [62] has explicitly suggested that the hippocampus may play a role in finding single states with which to fill in these intervening gaps, which may explain the hippocampal-dependence of trace-conditioning [55], [59]. Consistent with this, Pastalkova *et al*. [63] have found hippocampal sequences which step through intervening states during a delay period. Levy's theory predicted that it should take some time for that set of intervening states to develop [62]; before the system has settled on a set of intervening states, µAgents would distribute themselves among the large set of potential states, producing an equivalent-set-like effect. This hypothesis predicts that it should be possible to create intermediate versions of trace and delay conditioning by filling the gap with stimuli of varying predictive usefulness, thus effectively controlling the size of the set of equivalent states. The extant data seem to support this prediction [55], [64].

### The disappearance of CS-related dopamine signals with overtraining

During classical conditioning experiments, dopamine signals occur initally at the delivery of reward (which is presumably unexpected). With experience, as the association between the predictive cue stimulus (CS) and the reward (unconditioned stimulus, US) develops, the dopamine signal vanishes from the time of delivery of the US and appears at the time of delivery of the CS [34]. However, with extensive overtraining with very regular intertrial intervals, the dopamine signal vanishes from the CS as well [32].

Classical conditioning can be modeled in one of two ways: as a sequence of separate trials, in which the agent is restarted in a set 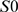 state each time or as a loop with an identifiable inter-trial-interval (ITI) state [5], [8], [14], [24]. While this continuous looped model is more realistic than trial-by-trial models, with the inclusion of the ITI state, an agent can potentially see across the inter-trial gap and potentially integrate the value across all future states. Eventually, with sufficient training, an agent would not show any 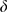 signal to the CS because there would be no unexpected change in value at the time the CS was delivered. We have found that this decrease happens very quickly with standard TD simulations (tens to hundreds of trials, data not shown). However, Ljungberg *et al*. report that monkeys required 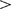
30,000 movements to produce this overtraining effect. This effect is dependent on strongly regular intertrial intervals (W. Schultz, personal communication).

The µAgents model suggests one potential explanation for the slowness of the transfer of value across the ITI state in most situations: Because the ITI state does not have a clearly identifiable marker, it should be encoded as a distributed representation over a large number of equivalent states. Presumably, in a classical conditioning task, the inter-stimulus interval is indicated by the presence of a strong cue (the tone or light). However, the appropriate cue to identify the inter-trial-interval (ITI) is not obvious to the animal, even though there are presumably many available cues. In our terminology, the ITI state forms a collection of *equivalent states*. Because all of these ITI states provide the same observation, the agent does not know which state the world entered and the µAgents distribute over the many equivalent ITI states. The effect of this is to distribute the 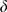 signal (and thus the change in value) over those many equivalent states. Thus the value of the ITI states remains low for many trials, and the appearance of an (unexpected) CS produces a change in value and thus a positive 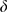 signal.

#### Simulations

**Figure 7:**
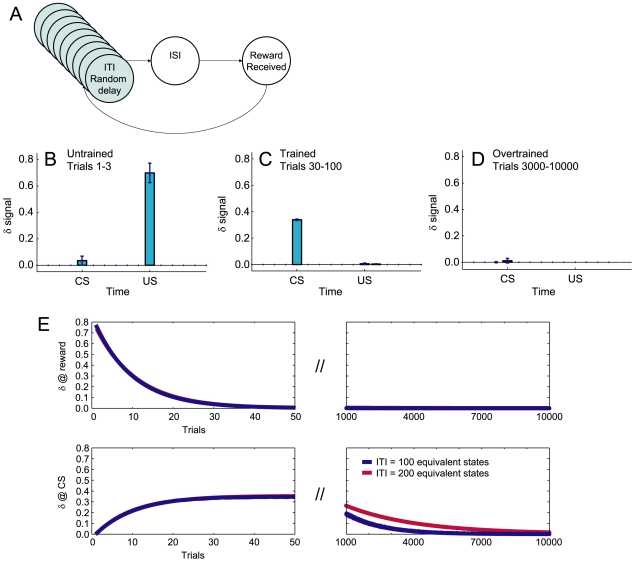
Effect of equivalent ITI states on δ signals at conditioned stimuli. (A) A state-space for classical conditioning. (B, C, D) Learning signaled reward delivery. (B) Untrained: *δ* occurs at US but not CS. (C) Trained: *δ* occurs at CS but not US. (D) Overtrained: *δ* occurs at neither CS nor US. (E–H) Transfer of value-error *δ* signal. Left panels show the first 50 trials, while right panels show trials 1000 to 10,000. *Y*-axes are to the same scale, but *x*-axes are compressed on the right panels. Increasing the number of equivalent ITI states increases the time to overtraining. Compare [32].

In order to test the time-course of overtraining, we simulated a standard classical conditioning task (Figure 7A). Consistent with many other TD simulations, the value-error 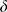 signal transferred from the reward to the CS quickly (on the order of 25 trials) (Figure 7B,C,E). This seemingly steady-state condition (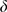 in response to CS but not reward) persists for hundreds of trials. But as the learned value-estimates of the equivalent ITI states gradually increase over thousands of trials, the 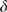 signal at the CS gradually disappears (Figure 7D,E). The ratio of time-to-learn to time-to-overlearn is compatible with the data of Ljungberg *et al*. [32]. Increasing the number of equivalent states in the ITI further slows abolition of 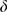 at the CS (Figure 7E).

#### Discussion and implications

The prediction that the inability of the delta signal to transfer across ITI states is due to the ITI state's lack of an explicit marker suggests that it should be possible to control the time course of this transfer by adding markers. Thus, if explicit, salient markers were to be provided to the ITI state, animals should show a faster transfer of delta across the ITI gap, and thus a faster decrease in the delta signal at the (no-longer-unexpected) CS. This also suggests that intervening situations without markers should show a slow transfer of the delta signal, as was proposed for trace conditioning above.

### Transient dopamine bursts at uncued movement initiation

Dopamine cues occurring at cue-stimuli associated with expected reward have been well-studied (and well-modeled) in Pavlovian conditioning paradigms. However, dopaminergic signals also appear just prior to uncued movements in instrumental paradigms [56], [65] and can appear even without external signals [57]. One potential explanation is that this dopamine signal is indicative of an internal transition occurring in the agent's internal world-model, perhaps from a state in which an action is unavailable to a state in which an action is available, thus providing a change in value and thus providing a small 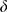 signal. Only a few µAgents would have to make this transition in order to produce such a signal and initiate an action. Once the action was initiated, the other µAgents would be forced to update their state belief in order to remain compatible with the ensuing world observations.

#### Simulations

**Figure 8:**
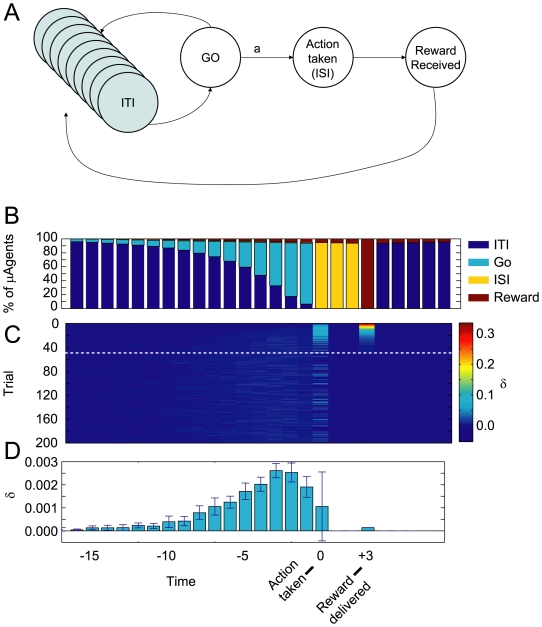
Modeling dopaminergic signals prior to movement. (A) State space used for simulations. The GO state has the same observation as the ITI states, but from GO an action is available. (B) Due to the expected dwell-time distribution of the ITI state, µAgents begin to transition to the GO state. When enough µAgents have their state-belief in the GO state, they select the action a, which forces a transition to the ISI state. After a fixed dwell time in the ISI state, reward is delivered and µAgents return to the ITI state. (C) As µAgents transition from ITI to GO, they generate *δ* signals because V(GO)>V(ITI). These probabilistic signals are visible in the time steps immediately preceding the action. Trial number is represented on the y-axis; value learning at the ISI state leads to quick decline of *δ* at reward. (D) Average *δ* signal at each time step, averaged across 10 runs, showing pre-movement *δ* signals. These data are averaged from trials 50–200, illustrated by the white dotted line in C. B, C, and D share the same horizontal time axis. Compare to [56].

In order to test the potential existence of dopaminergic signals just prior to movement appearing with no external cues, we built a state-space which contained an internally- but not externally-differentiated GO state (Figure 8A). That is, the GO-state was not identifiably different in the world, but actions were available from it. µAgents in the ITI state would occasionally update their state belief to the GO state due to the similarity in the expected observations in the GO and ITI states. If a sufficient number of µAgents were present in the GO state, the agent could take the action. Because the GO state was temporally closer to the reward than the ITI state, more value was associated with the GO state than with the ITI state. Thus, a µAgent transitioning into the GO state would produce a small 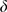 signal. Taking an action requires the overall agent to believe that the action is possible. However, there is no external cue to make the µAgents all transition synchronously to the GO state, so they instead transition individually and probabilistically, which produces small pre-movement 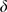 signals. In the simulations, µAgents gradually transitioned to the GO state until the action was taken (Figure 8B, top panel). During this time immediately preceding movement, small probabilistic 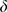 signals were observed (Figure 8B, middle panel). When these signals were averaged over trials, a small ramping 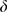 signal was apparent prior to movement (Figure 8B, bottom panel).

#### Discussion and implications

As can be seen in Figure 8, there is a ramping of delta signals as µAgents transfer from the ITI state to the GO state. A similar ramping has been seen in dopamine levels in the nucleus accumbens preceding a lever press for cocaine [56, e.g. Figure 2, p. 615]. This signal has generally been interpreted as a causative force in action-taking [65]. The signal in our simulation is not causative; instead it is a read-out of an internal shift in the distributed represented state of the macro-agent—the more µAgents there are in GO state, the more likely the macro-agent is to take action. Whether this ramping 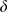 signal is a read-out or is causative for movement initiation is an open-question that will require more detailed empirical study.

### Other TD simulations

The µAgents model proposed here enabled novel explanations and models for (a) hyperbolic discounting, (b) differences between trace- and delay-conditioning, (c) effects of overtraining, and (d) the occurrence of dopamine signals prior to self-initiated movement.

However, TD models have been shown in the past to be able to accommodate a number of other critical experiments, including (e) that unsignaled reward produces a positive dopamine signal (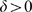) [5], [8], [18], [24], [32], [34], [66], [67], (f) that phasic dopamine signals (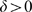) transfer from the time of an unconditioned stimulus to the time of the corresponding conditioning stimulus [1], [2], [8], [18], [19], [21], [32], [34], (g) that dopamine neurons pause in firing (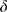 decreases) with missing, but expected, rewards [5], [8], [18], [24], [32]–[34], (h) that early reward produces a positive dopamine signal (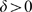) with no corresponding decrease at the expected reward time [5], [8], [24], [33], (i) that late reward produces a negative dopamine signal (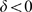) at the expected time of reward and a positive dopamine signal (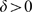) at the observed (late) reward [5], [8], [24], [33]. Finally, TD models have been able to explain (j) dopamine responses to changing probabilities of receiving reward [5], [8], [68], and (k) generalization responses [15], [34].

Extensive previous work already exists on how TD models capture these key experimental results. Some of these cases occur due to the basic identification of the phasic dopamine signal with 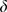 [1], [2], [11]. Some occur due to the use of semi-Markov models (which allows a direct simulation of time) [5], [8], [44]. Others occur due to the distributed representation of belief (e.g. *partially observability* [5], [8], [15], [44]). Because our µAgents model is an implementation of all of these, it also captures these basic results. Although the results included in this supplemental section do not require µAgents, the inclusion of µAgents does not lose them, which we briefly illustrate here.

#### Unsignaled reward produces a positive 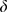 signal

**Figure 9:**
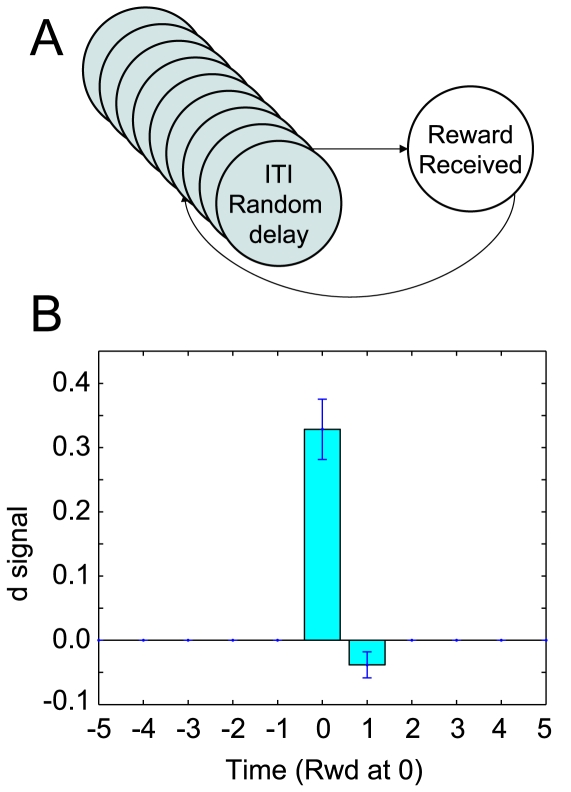
Unsignalled reward modulates δ. (A) State-space used for unsignaled reward. (B) *δ* increases at unexpected rewards.

When presented with an unexpected reward signal, dopamine neurons fire a short phasic burst [32], [34], [69]. Following Daw [8], this was modeled by a simple two state state-space: after remaining within the ITI state for a random time (drawn from a normal distribution, 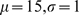 time-steps), the world transitioned to a *reward-state*, during which time a reward was delivered, at the completion of which, the world returned to the ITI state (Figure 9A). On the transition to the reward state, a positive 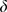 signal occurred (Figure 9B). Standard TD algorithms produce this result. Using sets of equivalent states to represent the ITI extends the time that the US will continue to cause a dopamine surge. Without this set of equivalent ITI states, the dopamine surge to the US would diminish within a number of trials much smaller than observed in experimental data.

#### 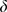 transfers from the unconditioned reward to conditioned stimuli

With unexpected reward, dopamine cells burst at the time of reward. However, when an expected reward is received, dopamine cells do not change their firing rate [32], [34]. Instead, the dopamine cells fire a burst in response to the conditioned stimulus (CS) that predicts reward [32], [34]. Following “the dopamine as 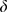
” hypothesis, this transfer of 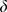 from reward to anticipatory cues is one of the keys to the TD algorithm [1], [2], [8], [34]. We modeled this with a three-state state-space (ITI, ISI, and Rwd; Figure 7A). As with other TD models, 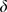 transferred from US to CS (Figure 7B,C,E). We modeled the ITI state as a set of equivalent states to extend the time that the CS will continue to cause a dopamine surge. In previous looped models, the dopamine surge to the CS would diminish within a small number of trials, giving a learning rate incompatible with realistic CS-US learning. As with other TD models living within a semi-Markov state-space [5], [8], the delta signal shifted back from the reward state to the previous anticipatory stimulus without progressing through intermediate times [70].

#### Missing, early, and late rewards

When expected rewards are omitted, dopamine neurons pause in their firing [18], [32], [33]. When rewards are presented earlier or later than expected, dopamine neurons show an excess of firing [33]. Importantly, late rewards are preceded by a pause in firing at the expected time of reward [33]. With early rewards, the data is less clear as to the extent of the pause at the time of expected reward (see Figure of Hollerman *et al*. [33]). As noted by Daw *et al*. [5] and Bertin *et al*. [24], these results are explicable as consequences of semi-Markov state-space models.

In semi-Markov models, the expected time distribution of the ISI state is explicitly encoded. µAgents will take that transition with the expected time distribution of the ISI state. These µAgents will find a decrease in expected value because no actual reward is delivered. The 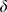 signal can thus be decomposed into two components: a positive 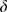 signal arising from receipt of reward and a negative signal arising from µAgents transitioning on their own. These two components can be separated temporally by providing reward early, late, or not providing it at all (missing reward).

**Figure 10:**
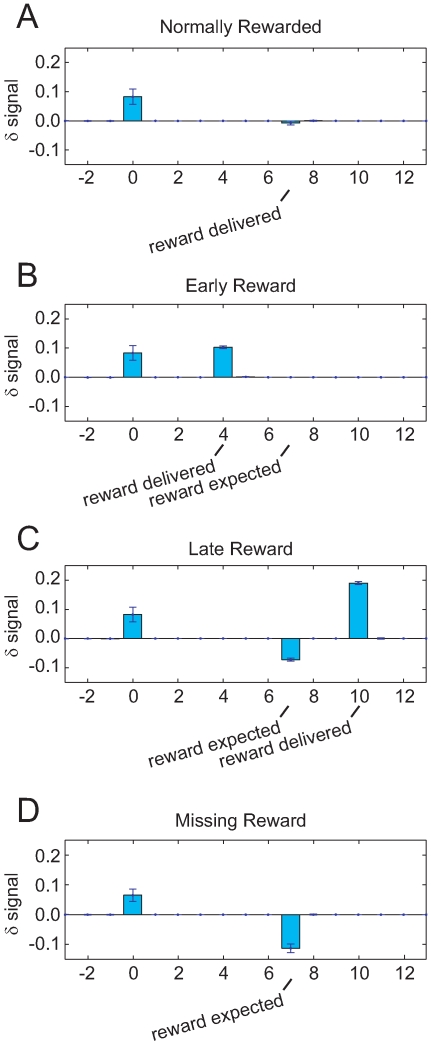
Early, late, and missing rewards modulate δ. (A) After training, *δ* is seen at CS but not US. (B) If reward is delivered early, *δ* appears at US. (C) If reward is delivered late, negative *δ* appears at the time when reward was expected, and positive *δ* occurs when reward is actually delivered. (D) If reward is omitted, negative *δ* occurs when reward was expected.

After training with a classical conditioning task, a 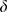 signal occurs at the CS but not the US (Figure 10A). When we delivered occasional probe trials on which reward arrived early, we observed a 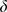 signal at the US (Figure 10B). This is because the value of the CS state accounts for a reward that is discounted by the normal CS-US interval. If the reward occurs early, it is discounted less. On the other hand, when we delivered probe trials with late reward arrival, we observed a negative 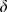 signal at the expected time of reward followed by a positive 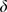 signal at the actual reward delivery (Figure 10C). The negative 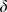 signal occurs when µAgents transition to the reward state but receive no actual reward. The observation of the ISI state is incompatible with µAgents' belief that they are in the reward state, so µAgents transition back to the ISI state. When reward is then delivered shortly afterwards, it is discounted less than normal and thus produces a positive 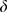 signal.

If reward fails to arrive when expected (missing reward), then the µAgents will transition to the reward state anyway due to their dwell-time and state hypotheses, at which point, value decreases unbalanced by reward. This generates a negative 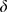 signal (Figure 10D). The signal is spread out in time corresponding to the dwell-time distribution of the ISI state.

#### 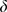 transfers proportionally to the probability of reward

**Figure 11:**
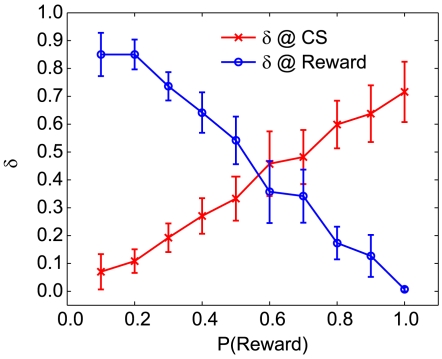
Probabilistic reward delivery modulates δ at CS and US. As the probability of reward drops, the *δ* signal shifts proportionately from the CS to the US. All measurements are taken after training for 100 trials.

TD theories explain the transfer seen in Figure 7 through changes in expected value when new information is received. Before the occurrence of the CS, the animal has no reason to expect reward (the value of the ITI state is low); after the CS, the animal expects reward (the value of the ISI state is higher). Because value is dependent on expected reward, if reward is given probabilistically, the change in value at the CS should reflect that probability. Consistent with that hypothesis, Fiorillo *et al*. [68] report that the magnitude of the dopamine burst at the CS is proportional to the probability of reward-delivery. In the µAgents model, a high probability of reward causes 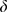 to occur at the CS but not US after training (Figure 11; also see Figure 7C and Figure 10A). As the probability of reward drops toward zero, 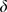 shifts from CS to US (Figure 11). This is because the value of the ISI state is less when it is not a reliable predictor of reward.

#### Generalization responses

When provided with multiple similar stimuli, only some of which lead to reward, dopamine neurons show a phasic response to each of the stimuli. With the cues that do not lead to reward, this positive signal is immediately followed by a negative counterbalancing signal [34]. As suggested by Kakade and Dayan [15], these results can arise from partial observability: on the observation of the non-rewarded stimulus, part of the belief distribution transfers inappropriately to the state representing a stimulus leading to a rewarding pathway. When that belief distribution transfers back, the negative 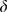 signal is seen because there is a drop in expected value. This explanation is compatible with the µAgents model presented here in that it is likely that some µAgents would shift to the incorrect state producing a generalization 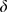 signal which would then reverse when those µAgents revise their state-hypothesis to the correct state.

**Figure 12:**
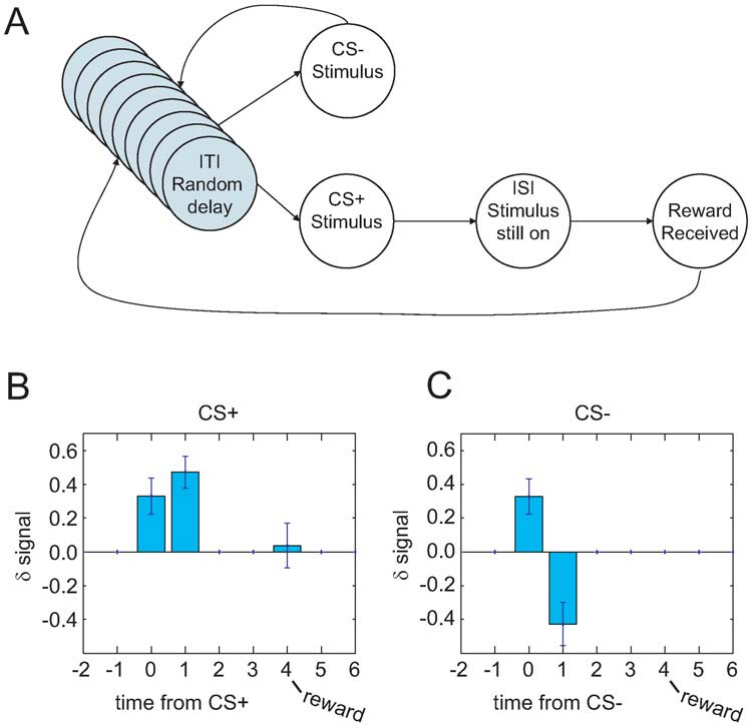
Effects of generalization on δ signals. (A) State-space used for measuring generalization. (B,C) Either CS+ or CS− produces a *δ* signal at time 0. (B) With CS+, the positive *δ* signal continues as µAgents transition to the ISI state, but (C) with CS−, the (incorrect) positive *δ* signal is counter-balanced by a negative *δ* correction signal when µAgents in the CS+ state are forced to transition back to the unrewarded ITI state.

To test the model's ability to capture the generalization result, we designed a state-space that contained two CS stimuli, both of which provided a “cue” observation. However, after one time-step, the CS- returned to the ITI state, while the CS+ proceeded to an ISI state, which eventually led to reward. Because (in this model), both the CS's provided similar observations, when either CS appeared, approximately half the µAgents entered each CS state, providing a positive 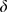 signal. In the CS- case, the half that incorrectly entered the CS+ state updated their state belief back to the ITI state after one time-step, providing a negative signal. In the CS+ case, the half that incorrectly entered the CS- state updated their state belief back to the ISI state after one time-step, providing a lengthened positive signal. See Figure 12.

## Discussion

In this paper, we have explored distributing two parameters of the standard temporal difference (TD) algorithm for reinforcement learning (RL): the discounting factor 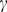 and the belief state 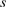. We implemented these distributed factors in a unified semi-Markov temporal-difference-based reinforcement learning model using a distribution of µAgents, the set of which provide a distributed discounting factor and a distributed representation of the believed state. Using distributed discounting produced hyperbolic discounting consistent with the experimental literature [40], [41]. The distributed representation of belief, along with the existence of multiple states with equivalent observations (i.e. *partial observability*), provided for the simulation of collections of “equivalent-states”, which explained the effects of overtraining [32], and differences between trace and delay conditioning [55]. Distributed state-belief also provided an explanation for transient dopamine signals seen at movement initiation [56], [57], as well as generalization effects [15].

Although the µAgents model we presented included both distributed discounting and distributed belief states (in order to show thorough compatibility with the literature), the two hypotheses are actually independent and have separable consequences.

### Distributed discounting

The mismatch between the expected exponential discounting used in most TD models and the hyperbolic discounting seen in humans and other animals has been recognized for many years [5], [8], [37], [38], [41], [71], [72].

Although hyperbolic discounting will arise from a uniform (and infinite) distribution of exponential functions [37, 73, see also Supporting Information *Appendix S1*], as the number of exponential functions included in the sum decreases, the discounting function deviates from true hyperbolicity. Changing the uniformity of the distribution changes the impulsivity of the agent (Figure 5). We also found that because the product of hyperbolic functions is not hyperbolic, it was necessary to maintain the separation of the discounting functions until action-selection, which we implemented by having each µAgent maintain its own internal value function 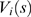 (Figure 4).

#### Other models

In addition to the suggestion that hyperbolic discounting could arise from multiple exponentials proposed here, three explanations for the observed behavioral hyperbolic discounting have been proposed [37]: (1) maximizing average reward over time [5], [71], [74], (2) an interaction between two discounting functions [75]–[77], and (3) effects of errors in temporal perception [8], [37].

While the assumption that animals are maximizing average reward over time [5], [71], [74] does produce hyperbolic discounting, assumptions have to be made that animals are ignoring intertrial intervals during tasks [37], [74]. Another complication with the average-reward theory is that specific dopamine neurons have been shown to match prediction error based on exponential discounting when quantitatively examined within a specific task [18]. In the µAgents model, this could arise if different dopamine neurons participated in different µAgents, thus recording from a single dopamine neuron would produce an exponential discounting factor due to recording from a single µAgent within the population.

The two-process model is essentially a two-µAgent model. While it has received experimental support from fMRI [76], [77] and lesion [78] studies, recent fMRI data suggest the existence of intermediate discounting factors as well [30]. Whether the experimental data is sufficiently explained by two exponential discounting functions will require additional experiments on very large data sets capable of determing such differences [45, see discussion in *Predictions*, below].

There is a close relationship between the exponential discounting factor and the agent's perception of time [8], [79], [80]. Hyperbolic discounting can arise from timing errors that increase with increased delays [79], [81], [82]. The duality between time perception and discounting factor suggests the possibility of a µAgent model in which the different µAgents are distributed over time perception rather than discounting factor. Whether such a model is actually viable, however, will require additional work and is beyond the scope of this paper.

### Distributed Belief

The concept of a distributed representation of the believed state of the world has also been explored by other researchers [5], [8], [23], [24], [83]. In all of these models (including ours), action-selection occurs through a probabilistic voting process. However, the 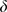 function differs in each model. In the Doya *et al*. [23] models, a single 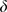 signal is shared among multiple models with a “responsibility signal”. In the Daw [5] models, belief is represented by a partially-observable Markov state process, but is collapsed to a single state before 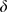 is calculated. Our distributed 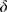 signal provides a potential explanation for the extreme variability seen in the firing patterns of dopaminergic neurons and in the variability seen in dopamine release in striatal structures [84], in a similar manner to that proposed by Bertin *et al*. [24].

#### Distributed attention

A multiple-agents model with distributed state-belief provides for the potential for situations represented as collection of equivalent states rather than as a single state. This may occur in situations without readily identifiable markers. For example, during inter-trial-intervals, there are many available cues (machinery/computer sounds, investigator actions, etc.) Which of these cues are the reliable differentiators of the ITI situations from other situations is not necessarily obvious to the animal. This leads to a form of divided attention, which we can model by providing the µAgents with a set of equivalent states to distribute across. While the µAgents model presented here requires the user to specify the number of equivalent states for a given situation, it does show that under situations in which we might expect to have many of these equivalent states, learning occurs at a slower rate than over situations in which there is only one state. Other models have suggested hippocampus may play a role in identifying unique states across these unmarked gaps [62], [63], [85]. While our model explains why learning occurs slowly across such an unmarked gap, the mechanisms by which an agent identifies states is beyond the scope of this paper.

The implementation of state representations used by many models are based on distributed neural representations. Because these representations are distributed, they can show variation in internal self-consistency—the firing of the cells can be consistent with a single state, or they can be distributed across multiple possibilities. The breadth of this distribution can be seen as a representation in the inherent uncertainty of the information represented [86]–[90]. This would be equivalent to taking the distribution of state belief used in the µAgents model to the extreme in which each neuron represents an estimate of a separate belief. Ludvig *et al*. [25], [26] explicitly presented such a model using a distributed representation of stimuli (“microstimuli”).

### Markov and semi-Markov state-spaces

Most reinforcement-learning models live within Markov state spaces (e.g. [1], [2], [67], [91], [92]), which do not enable the direct simulation of temporally-extended events. Semi-Markov models represent time explicitly, by having each state represent a temporally-extended event [5], [93]–[95].

In a Markov chain model, each state represents a single time-step, and thus temporally extended events are represented by a long sequence of states [93], [94], [96]. Thus, as a sequence is learned, the 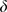 signal would step back, state by state. This backwards stepping of the 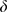 signal can be hastened by including longer eligibility traces [19] or graded temporal representations [25], [26], both of which have the effect of blurring time across the multiple intervening states. In contrast, in a semi-Markov model, each state contains within it a (possibly variable) dwell-time [5], [8], [93], [97], [98]. Thus while the 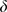 signal still jumps back state-by-state, the temporal extension of the states causes the signal to jump back over the full inter-stimulus time without proceeding through the intervening times. As noted by Wörgötter and Porr [70], this is more compatible with what is seen by Schultz and colleagues [13], [32], [34], [35], [99]–[101]: the dopamine signal appears to jump from reward to cue without proceeding through the intermediate times.

Semi-Markov state spaces represent intervening states (ISI states) as a single situation, which presumably precludes responding differently within the single situation. In real experiments, animals show specific time-courses of responding across the interval as the event approaches, peaking at the correct time [102]. The temporal distribution of dopamine neuron firing can also change across long delays [103]. Because our model includes a distribution of belief across the semi-Markov state space (the 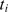 terms of the µAgent distribution), the number of µAgents that transition at any given time step can vary according to the distribution of expected dwell times. While matching the distributions of specific experiments is beyond the scope of this paper, if the probability of responding is dependent on the number of µAgents (Equation (5)), then the macro-agent can show a similar distribution of behavior (see Figure 8).

### Anatomical instantiations

The simulations and predictions reported here are based on behavioral observations and on the concept that dopamine signals prediction error. However, adding the hypotheses that states are represented in the cortex [5], [6], [104], while value functions and action selection are controlled by basal ganglia circuits [104]–[107] would suggest that it might be possible to find multiple µAgents within striatal circuits. Working from anatomical studies, a number of researchers have hypothesized that the cortical-striatal circuit consists of multiple separable pathways [27], [28], [108], [109]. Tanaka *et al*. [30] explicitly found a gradient of discounting factors across the striata of human subjects. This suggests a possible anatomical spectrum of discounting factors which would be produced by a population of µAgents operating in parallel, each with a preferred exponential discounting factor 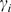. Many researchers have reported that dopamine signals are not unitary (See [8] for review). Non-unitary dopamine signals could arise from different dopamine populations contributing to different µAgents. Haber *et al*. [29] report that the interaction between dopamine and striatal neural populations shows a regular anatomy, in a spiral progressing from ventral to dorsal striatum. The possibility that Tanaka *et al*.'s slices may correspond to Haber *et al*.'s spiral loops, and that both of these may correspond to µAgents is particularly intriguing.

### Predictions

#### Hyperbolic discounting

The hypothesis that hyperbolic discounting arises from multiple exponential processes suggests that with sufficient data, the actual time-course of discounting should be differentiable from a true hyperbolic function. While the fit of real data to hyperbolic functions are generally excellent [39], [40], [48], [110], there are clear departures from hyperbolic curves in some of the data (e.g. [51], [54]). Rates of forgetting were also once thought to be hyperbolic [45], but with experiments done on very large data sets, rates of forgetting have been found, in fact, to be best modeled as the sum of multiple exponential processes [45], [46]. Whether discounting rates will also be better modeled as the sum of exponentials rather than as a single hyperbolic function is still an open question.

True hyperbolicity only arises from an infinite sum of exponentials drawn from a distribution with 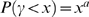, 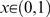. Under this distribution, the overall hyperbolic discounting is described by 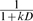, where 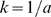. Changing the parameter 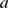 can speed up or slow down discounting while preserving hyperbolicity; changing the 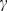 distribution to follow a different function will lead to non-hyperbolic discounting.

Serotonin precursors (tryptophan) can change an individual's discount rate [111], [112]. These serotonin precursors also changed which slices of striatum were active [112]. This suggests that the serotonin precursors may be changing the selection of striatal loops [29], slices [30], or µAgents. If changing levels of serotonin precursors are changing the selection of µAgents and the µAgent population contains independent value estimates (as suggested above), then learning under an excess of serotonin precursors may have to be relearned in the absence of serotonin precursors and vice-versa due to the change in the population of µAgents occurring with the change in serotonin levels.

In addition, in tasks structured such that exponential discounting maximizes the reward, subjects can shift their discounting to match the exponential to the task [113]. Drug-abusers [48], [50], smokers [51], [52], and problem gamblers [53] all show faster discounting rates than matched control groups. One possible explanation is that these altered overall discounting rates reflect differences in the distribution of µAgent discounting factors. As shown in Figure 5, biasing the µAgent 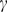 distribution can speed or slow overall discounting. Further, while a 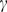 distribution following 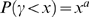 exhibits hyperbolic discounting, other distributions lead to non-hyperbolic discounting. Model comparison could be used on human behavioral data to determine if subsets of subjects show such patterns of discounting. However, this may require very large data sets [45].

#### Distributed belief and collections of equivalent states

The hypothesis that the slow development of overtraining [32] and the differences between trace- and delay conditioning [55] occur due to the distribution of attention across collections of equivalent states implies that these effects should depend on the ambiguity of the state given the cues. Thus, value should transfer across a situation proportionally to the identifiability of the that situation. Decreasing cue-ambiguity during inter-trial-intervals should speed up the development of overtraining (observable as a faster decrease in dopamine signal at the CS). Increasing cue-ambiguity during inter-stimulus-intervals should slow down learning rates of delay-conditioning. As the cues become more ambiguous and less salient, delay-conditioning should become closer and closer to trace conditioning. The extant data seem to support this prediction [55], [64].

### Summary/Conclusion

In this paper, we explored distributing two parameters of temporal difference (TD) models of reinforcement learning (RL): distributed discounting and distributed representations of belief. The distributed discounting functions provide a potential mechanistic explanation for hyperbolic discounting. The distributed representations of belief provide potential explanations for the decrease in dopamine at the conditioned stimulus seen in overtrained animals, for the differences in learning rate between trace and delay conditioning, and for transient dopamine at movement initiation. These two hypotheses, although separable, together provide a unified model of temporal difference reinforcement learning capable of explaining a large swath of the experimental literature.

## Supporting Information

Appendix S1 Hyperbolic discounting can arise from a sum of exponentials.
(0.03 MB PDF)
