# Correction: Temporal-Difference Reinforcement Learning with Distributed Representations

**DOI:** 10.1371/annotation/4a24a185-3eff-454f-9061-af0bf22c83eb

**Published:** 2009-12-11

**Authors:** Zeb Kurth-Nelson, A. David Redish

The initials for author A. David Redish appear incorrectly in the Author Contributions. The correct initials are ADR. The Author Contributions should read: "Conceived and designed the experiments: ZKN DDR. Performed the experiments: ZKN ADR. Analyzed the data: ZKN ADR. Wrote the paper: ZKN ADR."

