# Supplementary material for: Temporal-Difference Reinforcement Learning with Distributed Representations
Source: PLoS One. 2009 Oct 20;4(10):e7362. doi: 10.1371/journal.pone.0007362 (PMC2760757; doi:10.1371/journal.pone.0007362)
Supplement: Appendix S1 — Hyperbolic discounting can arise from a sum of exponentials. (0.03 MB PDF) [file pone.0007362.s001.pdf]

Temporal-difference reinforcement learning with distributed representations

Zeb Kurth-Nelson, A. David Redish

Appendix S1

Hyperbolic discounting from a sum of exponentials. The summed effect of these exponential discounting functions provides the overall agent with hyperbolic discounting:

$$\int_0^1 \gamma^x d\gamma = \frac{1}{1+x} \tag{1}$$

By the standard integration power law,

$$\int_0^1 \gamma^x d\gamma = \lim_{\gamma \to ^+ 0} \frac{-(\gamma^{x+1} - 1)}{x+1} \tag{2}$$

which, if $x > 0$, approaches $1/(1+x)$ as γ approaches 0 from $\gamma > 0$.
